# Distribution and mixing of old and new nonstructural carbon in two temperate trees

**DOI:** 10.1111/nph.13273

**Published:** 2015-01-05

**Authors:** Andrew D Richardson, Mariah S Carbone, Brett A Huggett, Morgan E Furze, Claudia I Czimczik, Jennifer C Walker, Xiaomei Xu, Paul G Schaberg, Paula Murakami

**Affiliations:** 1 Department of Organismic and Evolutionary Biology, Harvard UniversityCambridge, MA, 02138, USA; 2 Earth Systems Research Center, University of New HampshireDurham, NH, 03824, USA; 3 Department of Biology, Bates CollegeLewiston, ME, 04240, USA; 4 Department of Earth System Science, University of CaliforniaIrvine, CA, 92697-3100, USA; 5 USDA Forest Service, Northern Research StationBurlington, VT, 05405, USA

**Keywords:** carbohydrates, carbon allocation, Harvard Forest, radiocarbon (^14^C), storage, tree rings, wood

## Abstract

- We know surprisingly little about whole-tree nonstructural carbon (NSC; primarily sugars and starch) budgets. Even less well understood is the mixing between recent photosynthetic assimilates (new NSC) and previously stored reserves. And, NSC turnover times are poorly constrained.
- We characterized the distribution of NSC in the stemwood, branches, and roots of two temperate trees, and we used the continuous label offered by the radiocarbon (carbon-14, ^14^C) bomb spike to estimate the mean age of NSC in different tissues.
- NSC in branches and the outermost stemwood growth rings had the ^14^C signature of the current growing season. However, NSC in older aboveground and belowground tissues was enriched in ^14^C, indicating that it was produced from older assimilates. Radial patterns of ^14^C in stemwood NSC showed strong mixing of NSC across the youngest growth rings, with limited ‘mixing in’ of younger NSC to older rings.
- Sugars in the outermost five growth rings, accounting for two-thirds of the stemwood pool, had a mean age < 1 yr, whereas sugars in older growth rings had a mean age > 5 yr. Our results are thus consistent with a previously-hypothesized two-pool (‘fast’ and ‘slow’ cycling NSC) model structure. These pools appear to be physically distinct.

## Introduction

Although plant photosynthesis itself is well understood, much less is known about processes regulating the allocation of photosynthetic assimilates to growth, storage, and other metabolic functions (Wiley & Helliker, 2012; Dietze *et al*., 2014). Storage of nonstructural carbon (NSC, principally sugars and starch; Hoch *et al*., 2003) is critically important for woody plants, because these reserves enable sessile, long-lived organisms to tolerate biotic and abiotic stress, including pests, disturbance, and drought (Körner, 2003; Dietze *et al*., 2014). Thus, NSC reserves are highly relevant in the context of tree resilience to many global change factors. But, critical questions about the size and turnover of these reserves remain unanswered (Carbone *et al*., 2013; Muhr *et al*., 2013; Richardson *et al*., 2013; Dietze *et al*., 2014).

Isotope labeling experiments have provided key insights into the use of NSC to support tree growth and metabolism (Epron *et al*., 2012). This work has conclusively shown that new tissue growth typically relies on some mixture of both ‘old’ (previously stored) and ‘new’ (recent photosynthetic assimilates) NSC, although the reliance on stored reserves varies depending on the tissue in question, the time of year, and also among species (Kagawa *et al*., 2006; Keel *et al*., 2006, 2007; Carbone *et al*., 2007; Kuptz *et al*., 2011; Krepkowski *et al*., 2013). For logistical reasons, few labeling studies have been conducted on large, mature trees (Keel *et al*., 2006; Carbone *et al*., 2007). And, while previous studies have provided important insight into the short-term (hours–months) patterns of carbon (C) allocation and use, the long-term (years–decades) fate of the labeled C in structural and nonstructural pools has not been characterized (Kagawa *et al*., 2006; Keel *et al*., 2006; Högberg *et al*., 2008).

The radiocarbon (carbon-14, ^14^C) bomb spike (Levin & Kromer, 2004) provides an alternative to conventional isotope labeling for tracking the long-term fate of assimilated CO_2_ (Gaudinski *et al*., 2001). Using this method, we previously identified NSC more than a decade old in the stemwood of two temperate tree species (Richardson *et al*., 2013). Other work has shown that stored, decade-old C can be used to regrow root (Vargas *et al*., 2009) and shoot (Carbone *et al*., 2013) tissues following major disturbance. These are surprising results, given that short-term pulse-chase labeling studies have generally shown rapid use of new (labeled) NSC and inferred fast mixing between old and new NSC, both of which suggest quick turnover of storage reserves (Keel *et al*., 2007; Von Felten *et al*., 2007; Krepkowski *et al*., 2013).

Because whole-tree budgets of NSC are also scarce, we have few data with which to constrain estimates of NSC pool sizes and turnover times (Körner, 2003; Trumbore, 2006; Gaudinski *et al*., 2009; Muhr *et al*., 2013). Thus, to better understand how NSC reserves are distributed, the degree to which these reserves represent a mix of older vs newer NSC, and how this mixing varies throughout the tree, we destructively sampled a 23-yr-old white pine (*Pinus strobus*) and a 30-yr-old red oak (*Quercus rubra*) from Harvard Forest, an oak-dominated temperate forest in the north-eastern Unites States. This study thus builds on our previous work (Carbone *et al*., 2013; Richardson *et al*., 2013) by constructing whole-tree NSC budgets and by examining the radial patterns in NSC concentration and age, which allow us to directly examine the evidence for different mixing scenarios.

**Figure 1:**
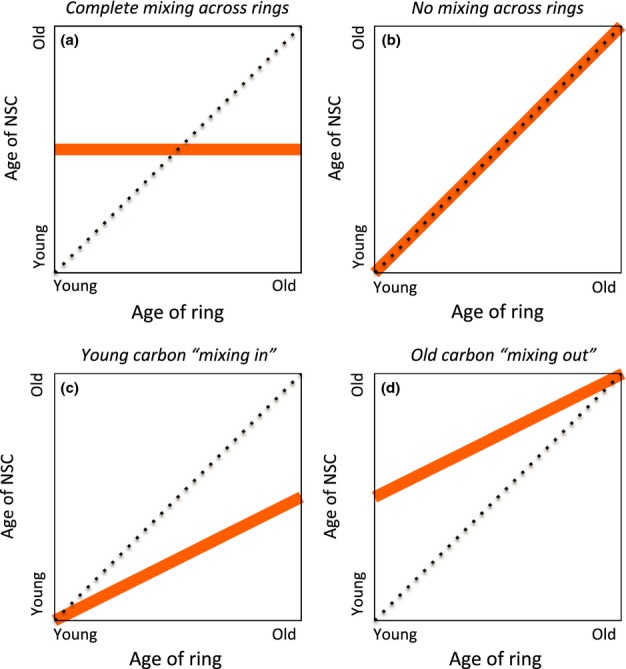
Conceptual models indicating possible scenarios for mixing of nonstructural carbon (NSC) across tree ring boundaries. In the case where there is complete mixing of ‘old’ (stored) NSC and ‘new’ (recent photosynthetic assimilates) NSC across the radial profile, the measured age of extracted NSC should be constant with depth, that is, across rings of different ages (a). When there is no mixing of old and new, and NSC is effectively ‘locked’ into the ring in which it is first deposited, then the measured age of extracted NSC should increase in parallel with ring age (b). However, if younger NSC ‘mixes in’ to older rings, the age of NSC should always be younger than the ring age (c), whereas if older NSCs ‘mixes out’ to younger rings, the age of NSC should always be older than the ring age (d). Intermediate cases are possible, for example, partial mixing would be characterized by young rings where the NSC is older than the ring age, and old rings where the NSC is younger than the ring age. The dashed line indicates the 1:1 line, and the orange line indicates the mixing scenario.

We analyzed stemwood samples (divided into individual rings, bark, and phloem), current- and multi-year branches, and three different root diameter size classes, for concentrations of total sugars and starch, and integrated these to whole-tree budgets using allometric scaling (Jenkins *et al*., 2004). Then, by comparing the age of stored NSC (estimated via ^14^C analysis) to the age of the tissue from which it was extracted, we evaluated four candidate conceptual models for the mixing of old and new NSC (Fig.1). The ‘complete mixing’ model is what is implicitly assumed in most contemporary ecosystem and tree growth models that include a storage pool (Riley *et al*., 2009; Richardson *et al*., 2013; Dietze *et al*., 2014), whereas the limited evidence to date is more consistent with the ‘no mixing’ model (Taylor *et al*., 2007). Our previous work has been unable to distinguish between these models (Carbone *et al*., 2013; Richardson *et al*., 2013).

## Materials and Methods

### Study site

We conducted field sampling in November 2012 within the Tom Swamp (42°30.8′N, 72°13.1′W) tract of the Harvard Forest, *c*. 110 km west of the city of Boston, MA, USA. The mean annual temperature is 6.5°C, and mean annual precipitation is 1000 mm, distributed evenly throughout the year. The soil is a well-drained sandy loam derived from glacial till. The stand was previously a red pine (*Pinus resinosa* Ait.) plantation that was thinned in 1983 and harvested in 1990. The trees we sampled regenerated naturally and are young enough to contain no pre-bomb (before ad 1950) C. The forest in the vicinity of our sampling site is dominated by the two study species, white pine (*Pinus strobus* L.) and red oak (*Quercus rubra* L.). These species were selected primarily because the annual growth rings in stemwood disks were conspicuous and could be clearly identified by the naked eye, with no sanding or other preparation required. White pine is an evergreen conifer, while red oak is a ring porous, deciduous hardwood. Both species are of intermediate shade tolerance, have a wide distribution across the eastern half of North America, and are reproductively mature by *c*. 25 yr of age.

### Field collection

We selected one tree of each species for destructive harvesting. The cost of conducting the ^14^C analyses precluded the sampling of additional trees. The main criteria for selection were (1) evidence of vigorous growth and (2) a well-developed crown that was not overtopped by surrounding trees. The pine was 12 m high, 23 yr of age, 18.5 cm diameter at breast height (DBH), and established following the harvest in 1990. The oak was 20 m high, 30 yr of age, and 17.0 cm DBH, and established following the thinning in 1983. Trees were felled and stemwood bolts (*c*. 50 cm in length) collected at breast height, mid-way up the stem, and near the top of the stem. We also collected current-year twigs, as well as multi-year branches, from throughout the crown. We then partially excavated the stump and root system of each tree, and collected samples of large coarse roots (*ϕ* > 5 cm), as well as medium coarse roots (*ϕ* ≈ 1 cm) and fine roots (*ϕ* ≤ 1 mm) that were directly attached to the large coarse roots (and thus undoubtedly from the same tree).

### Laboratory preparations

Samples were processed in the laboratory on the same day that they were collected in the field. Cookies, or stemwood sections *c*. 2 cm in thickness, were cut from the stemwood bolts. Using a hammer and chisel, we separated the outer bark and phloem, and then separated individual annual growth rings from each cookie. We identified the heartwood–sapwood transition based on wood color.

In the large and intermediate coarse roots we could not easily count rings. We therefore separated the large coarse root wood by thirds into three depths, with D1 denoting the outer (most recent) wood and D3 the center (oldest) wood. For all root samples, in lieu of ring counts we determined the mean age of the cellulose (i.e. structural C) via ^14^C analysis as described later.

Samples were frozen at −80°C and then freeze-dried (FreeZone 2.5; Labconco, Kansas City, MO, USA, and Hybrid Vacuum Pump, Vacuubrand, Wertheim, Germany) before being homogenized and milled to 20 mesh (Wiley Mini Mill; Thomas Scientific, Swedesboro, NJ, USA). Samples were then kept at −80°C until the NSC extraction was conducted. Additional sample material was kept frozen at −80°C.

### NSC analysis

For total soluble sugar concentration (following Chow & Landhäusser, 2004), 50 mg of tissue was freeze-dried a second time (24 h; for determination of precise weight) and subjected to hot ethanol extraction followed by colorimetric analysis with phenol-sulfuric acid. The resulting extract was read at 490 nm with a microplate reader (Epoch Microplate Spectrophotometer; Bio-Tek Instruments, Winooski, VT, USA) with sugar concentration (expressed as milligrams of sugar per gram of dry wood) calculated from a standard curve of 1:1:1 glucose–fructose–galactose (Sigma Chemicals, St Louis, MO, USA).

For starch analysis (following Wargo *et al*., 2002), the remaining tissue residue was boiled in potassium hydroxide (KOH) followed by neutralization with acetic acid and enzymatic digestion with amyloglucosidase. Glucose hydrolyzate was determined using a glucose hexokinase kit (Pointe Scientific, Canton, MI, USA) and read at 340 nm with the microplate reader. Starch concentration (expressed as milligrams of starch per gram of dry wood) was calculated based on glucose standard (Pointe Scientific, Canton, MI, USA) curves.

The NSC concentration data for each sample are reported in Supporting Information Table S1.

### ^14^C analysis

Following Richardson *et al*. (2013) and Carbone *et al*. (2013) we used the ^14^C bomb spike to estimate the age of extracted NSC, and in the case of root wood, which could not be easily aged by ring counting, to estimate the age of the cellulose (i.e. structural C). The bomb spike method is based on the fact that aboveground testing of thermonuclear weapons between 1955 and 1963 approximately doubled the amount of ^14^CO_2_ in the atmosphere (Levin & Kromer, 2004). Since 1963, when the Limited Nuclear Test Ban Treaty was signed, the ^14^C content of atmospheric CO_2_ has decreased through mixing with oceanic and terrestrial C reservoirs, and by addition of ^14^C-free CO_2_ from fossil fuel burning (Levin *et al*., 2010). The C in photosynthetic products reflects the ^14^C content of atmospheric CO_2_ in the year assimilation occurred, and hence these products are labeled with a unique isotopic signature. Thus, the mean ^14^C age of a sample can be determined by measuring its ^14^C content, and comparing this to the atmospheric ^14^CO_2_ record.

We conducted ^14^C analysis on sugars and starch extracted following Czimczik *et al*. (2014). Soluble NSC (mostly sugars) was isolated by hot water extraction, and insoluble NSC (mostly starch) was isolated by acid digestion following lipid removal by boiling in ethanol. The extractions were conducted sequentially from each tissue sample. We conducted the ^14^C analysis on cellulose extracted using a Soxhlet apparatus (Leavitt & Danzer, 1993).

For the ^14^C analysis of both NSC and cellulose, extracts were combusted to CO_2_, purified on a vacuum line, and converted to graphite (Xu *et al*., 2007). Graphite was analyzed for its ^14^C content at the W.M. Keck Carbon Cycle Accelerator Mass Spectrometry facility at University of California, Irvine (Southon *et al*., 2007).

We used the northern hemisphere atmospheric record (Levin *et al*., 2008) for dating, following Gaudinski *et al*. (2001). Previous ^14^CO_2_ samples (S. Trumbore, unpublished; Carbone *et al*., 2013; Richardson *et al*., 2013) have shown that the background air at Harvard Forest is consistent with the atmospheric record. For further verification, we have since 2010 collected annual plant samples (Jewelweed, *Impatiens capensis* Meerb. and ragweed, *Ambrosia artemisiifolia* L.) each year. Annual plants are natural ‘isometers’ because the ^14^C content in their structural tissues reflects an average daytime ^14^CO_2_ value of the atmosphere, integrated over weeks-to-months, for the current growing season. Because they live for only 1 yr, they have no stored NSC that could be carried over from previous years, and any previous-year seed signal is overwhelmed by current-year assimilation.

The ^14^C data for each sample are reported in Supporting Information Table S2. An analysis of the uncertainty in the ^14^C measurements is contained in Supporting Information Methods S1.

### Allometric scaling from concentrations to whole-tree budgets

We sanded stemwood disks, from breast height, using progressively finer sandpaper until all ring boundaries could be precisely identified under a dissecting microscope (Stereozoom; Leica Microsystems, Wetzlar, Germany). We measured ring widths (mean of three radii for pine, four radii for oak) under the microscope, using a sliding stage and linear encoder (TA Tree Ring System; Velmex Inc., Bloomfield, NY, USA) with a resolution of 0.001 mm and accuracy of 0.010 mm m^−1^. With these data, we could then estimate the stem biomass attributable to each year of growth (from the current growing season, 2012, to the first year at which each tree reached breast height – 1990 for the pine, and 1983 for the oak) using standard allometric scaling theory (Whittaker *et al*., 1974). For this we used equations and coefficients presented by Jenkins *et al*. (2004) to estimate stemwood, branch, and root biomass (in kilograms of dry wood).

We paired our tissue-specific (or in the case of stemwood rings, ring-specific) concentration measurements with our estimate of the woody biomass in each tissue type (or ring). We then determined the total amount (= biomass × concentration) of sugars and starch in each biomass component (stemwood, roots, and branches), and added these together to estimate the aggregate, whole-tree NSC pool stored in woody tissues. For additional details, see Supporting Information Methods S2.

## Results and discussion

**Figure 2:**
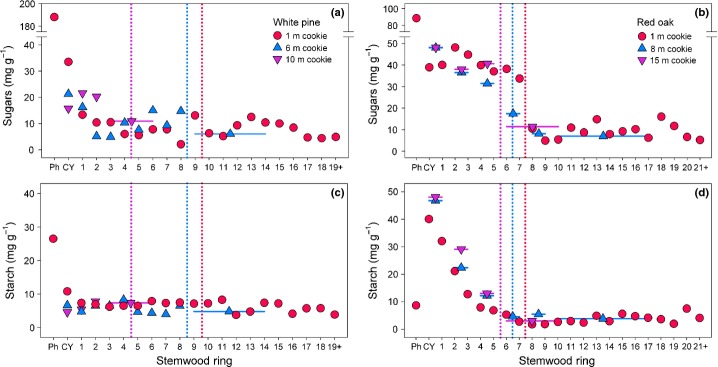
Radial patterns in stemwood concentrations of sugars (a, b) and starch (c, d) for white pine (*Pinus strobus*, a, c) and red oak (*Quercus rubra*, b, d). Samples were collected at three different heights in the tree. The vertical dotted lines indicate the sapwood–heartwood boundary at these heights, with the color corresponding to the sampling height. Thin horizontal lines indicate the range of rings included, for cases where multiple rings were pooled to produce a single sample for analysis. Ph is phloem tissue, and CY is the current-year (2012) ring.

In the stemwood, radial patterns of NSC concentrations differed between the two trees, although the highest concentrations of both sugars and starch were always observed in the phloem and/or outermost rings (Fig.2) (Hoch *et al*., 2003; Niamké *et al*., 2011). And, the degree to which the sapwood/heartwood transition was related to the radial patterns differed between trees (Barbaroux & Bréda, 2002). At 1 m height, concentrations of sugars in the pine dropped off rapidly within the first few rings (Fig.2a), whereas starch concentrations were low in all rings (Fig.2c). In the oak, concentrations of sugars were high until the sapwood/heartwood boundary (Fig.2b), and low thereafter, whereas starch concentrations declined steeply until the sapwood/heartwood boundary (Fig.2d). Similar patterns were observed for samples from the middle and top of the stem (Fig.2a–d).

In large coarse roots, NSC concentrations did not vary with radial depth in either species (Table S3). However, large and intermediate coarse root concentrations of both sugars and starch were generally much lower in the pine than the oak. By comparison, fine root concentrations of both sugars and starch were about equal in pine and oak.

In branches, concentrations of sugars and starch were lower in pine than oak (Table S3). In both trees, branch concentrations of sugars were higher than those of starch, particularly in current-year branches.

Scaled up to whole-tree budgets for NSC stored in woody tissues, we observed substantial differences between trees in total NSC, and how this total was partitioned between aboveground and belowground tissues (Table1; see also Tables S4–S6). For example, although the estimated whole-tree woody biomass of the pine (103 kg) was only 30% smaller than that of the oak (144 kg), the oak stored nearly four times as much NSC in its woody tissues. Thus, in the pine, NSC reserves were estimated at 1.5 kg of sugars and 1.1 kg of starch, compared with 4.8 kg of sugars and 5.3 kg of starch in the oak. In both trees, stemwood was the largest reserve of sugars, and roots the largest reserve of starch, but there was only one-eighth as much starch in the pine's roots (0.5 kg, 45% of the total starch pool) compared to the oak's roots (3.9 kg, 73% of the total starch pool). Furthermore, in both trees, the amount of NSC stored in the heartwood of the stem was relatively small: it accounted for < 15% of the stemwood total in the pine, and < 7% of the stemwood total in the oak. However, since parenchyma tissue in the heartwood is by definition dead, these reserves are therefore likely unavailable to the tree (Spicer, 2005). Finally, this scaling showed that branches are an important store of sugars, accounting for about one-quarter of the total stored in woody tissues, in both trees. Thus this analysis highlights the importance of employing a relatively detailed accounting scheme – including roots and branches – for scaling up to whole-tree NSC budgets (Gholz & Cropper, 1991; Barbaroux *et al*., 2003; Würth *et al*., 2005).

**Table 1:** Whole-tree budgets for nonstructural carbon (NSC) reserves stored in the woody tissues of a 23-yr-old white pine (*Pinus strobus*, top) and 30-yr-old red oak (*Quercus rubra,* bottom)

Component	Woody biomass (kg)	NSC content (g)		Per cent of tree total		Effective mean concentration (mg g^−1^)	
		Sugars	Starch	Sugars	Starch	Sugars	Starch
*White pine*							
Stem	60	644	428	42	38	11	7
Root	24	460	501	30	45	19	21
Branches	19	427	185	28	17	23	10
Total	103	1531	1114	100	100	15	11
*Red oak*							
Stem	71	2115	997	44	19	30	14
Root	30	1638	3897	34	73	56	132
Branches	44	1075	426	22	8	25	10
Total	144	4828	5320	100	100	33	37

The whole-tree budget was determined by scaling up from concentration measurements using allometric scaling theory (see the Materials and Methods section and Supporting Information Methods S2), as detailed in Table S4 (stemwood), Table S5 (roots), and Table S6 (branches).

**Figure 3:**
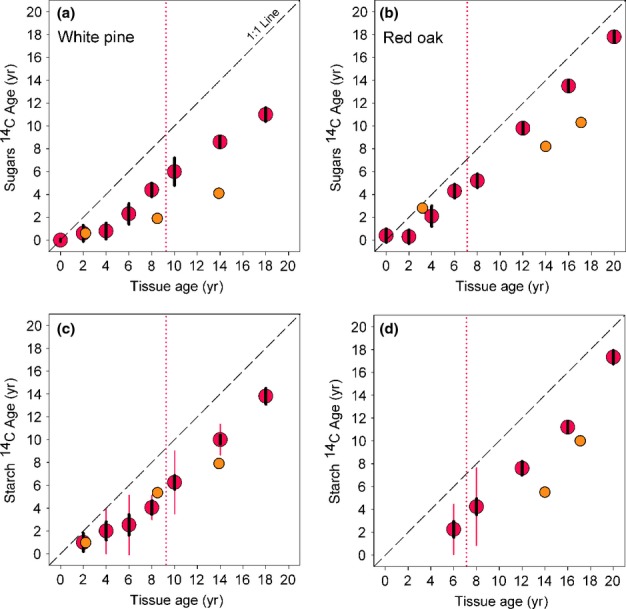
Radial trends in radiocarbon (^14^C) age (see the Materials and Methods section) of extracted sugars (a, b) and starch (c, d) in stemwood and large coarse root samples of white pine (*Pinus strobus*, a, c) and red oak (*Quercus rubra*, b, d). For stemwood, tissue age was determined by direct ring counts. For roots, tissue age was determined by ^14^C analysis of extracted cellulose (see the Materials and Methods section). The vertical dotted line indicates the sapwood–heartwood boundary at breast height. The thin red vertical error bars indicate the standard deviation of ^14^C age across repeat samples (where available; see Supporting Information Methods S1). The heavier black vertical bars indicate the analytical uncertainty (one standard deviation, 1 SD) of the accelerator mass spectrometry (AMS) measurements (see Methods S1).

In both pine and oak, and for both sugars and starch, NSC was always the same age or younger than the structural tissue from which it was extracted (Fig.3; Table S3). Notably, in the stemwood of both species, sugars in the outermost few rings were comprised primarily of current-year photosynthate: from the current-year ring to the 4-yr-old ring in pine, and to the 2-yr-old ring in oak, the mean age of extracted sugars in each ring was ≤ 1 yr. However, beyond these outermost rings, there was a pattern of sugars increasing steadily in age with increasing ring age. For pine, the age of sugars increased linearly (*r* = 0.98, slope = 0.75 ± 0.05 yr per ring, *n* = 5) from 2.3 yr in the 6-yr-old ring, to 11.0 yr in the 18-yr-old ring (Fig.3a). By comparison, in oak, the age of sugars also increased linearly (*r* = 0.99, slope = 0.98 ± 0.03 yr per ring, *n* = 6) but at a faster rate, from 2.1 yr in the 4-yr-old ring, to 17.8 yr in the 20-yr-old ring (Fig.3b). In both species, these data suggest strong mixing of relatively new sugars across the outermost rings (Fig.1a). However, it appears that mixing is more limited beyond about the 5-yr-old ring. There is no evidence in the stemwood data for old NSC ‘mixing out’ (Fig.1d), but in the case of pine, the data are consistent with some younger NSC ‘mixing in’ to older rings (Fig.1c), whereas in oak the data suggest much more limited mixing of younger NSC into older rings (Fig.1b). These results are surprising because oak wood has large and prominent ray cells (accounting for > 25% of wood volume; Brown *et al*., 1949), which might be expected to facilitate radial mixing, compared to the extremely small ray cells in pine (accounting for only 5% of wood volume; Brown *et al*., 1949). The higher total NSC amount and the apparently more limited mixing of younger NSC into older stemwood rings in the hardwood (deciduous oak) than the softwood (evergreen pine) reinforces the idea that wood anatomy and leaf habit are key drivers governing NSC allocation and availability (Hoch *et al*., 2003). Disturbance ecology could also play a role, in that red oak can resprout vigorously from the stump or root collar, whereas white pine does not resprout and so maintaining large reserves of NSC in the root system would be of limited value.

Overall, because the stem biomass is dominated by more recent growth rings (Table S4), which contain high concentrations of very young NSC, the weighted mean age (across all rings) of stemwood sugars was 2.2 yr in the pine and 2.4 yr in the oak. This mean age of stemwood sugars is younger than we previously reported for NSC extracted from 2 cm stemwood cores of red maple and eastern hemlock (Richardson *et al*., 2013). However, for the red maple in that analysis, there was a significant positive correlation (*r* = 0.66, *P* < 0.001, *n* = 26) between the age of extracted sugars (range: 2–24 yr) and tree age (range 70–194 yr). Consequently, the most likely reason we obtained younger ages here was because we sampled younger trees.

Additionally, we previously reported that stemwood NSC is highly dynamic on seasonal timescales, but also (on average) substantially older (mean ± 1 SD: 11 ± 7 yr for sugars + starch) than expected or predicted by most simulation models (Richardson *et al*., 2013). We resolved this apparent paradox of a dynamic, but old, pool by hypothesizing that there is not a single NSC pool, but rather two pools with functionally different roles: a ‘fast’ pool that turns over quickly and is preferentially used to support growth and metabolism, and a ‘slow’ pool, comprised of older NSC, that can be drawn on if reserves in the fast pool drop too low. The data presented here for stemwood sugars are consistent with this hypothesis: in both trees, the outermost five (current year to 4-yr-old) rings contain about two-thirds of the stemwood sugars, and together have a weighted mean age of < 1 yr, while the remaining one-third of stemwood sugars are found deeper in the stem and have a mean age of > 5 yr. Overall, the weighted mean age is thus 2–3 yr, but with this interpretation the ‘fast’ and ‘slow’ cycling components are stored in different rings. NSC in the outermost rings is likely considerably more accessible to the tree than NSC in deeper rings. But, previous studies have shown that old reserves are available to support growth following major disturbance (Vargas *et al*., 2009; Carbone *et al*., 2013).

The starch age data are not as complete because some samples were lost in processing, but they are consistent with the patterns for sugars, in that starch age increased steadily with radial depth and was always younger than the tissue from which it was extracted (Fig.3c,d). Pooling stemwood data for both species shows a strong linear relationship (*r* = 0.97; intercept not significantly different from zero, slope not significantly different from one, both *P* > 0.50 by *t*-test, *n* = 12) between the age of extracted sugars and the age of extracted starch. This is consistent with previous observations (Richardson *et al*., 2013) indicating seasonal cycles of substantial interconversion of sugar and starch in the stemwood of a variety of different temperate tree species. However, we interpret the radial patterns of NSC ages (Fig.3) to suggest that this mixing does not cross ring boundaries, except for possibly within the outermost few rings.

In roots, we found both young (< 1 yr) and old (> 5 yr) NSC, again suggesting poor mixing and possibly the presence of C reserves that turn over on different timescales (Table S3). But whereas in the stemwood old NSC was found only in older rings, where concentrations were low, old NSC was found at relatively high concentrations in the medium coarse roots and fine roots of pine, and in all roots of oak (Table S3). As was the case aboveground, NSC age in roots increased with increasing tissue age, and in large coarse roots with increasing radial depth. And, especially in older tissues, both sugars and starch tended to be substantially younger than the tissues from which they were extracted, which is consistent with the ‘mixing in’ of newer NSC (Fig.3). But, unlike what we found aboveground, the age of root sugars was poorly correlated with the age of root starch (*r* = 0.53, *P* = 0.17, *n* = 8), suggesting that less seasonal interconversion of sugars and starch occurs belowground, compared to aboveground.

### Conclusions

By using radiocarbon (^14^C) methods, this study has provided unique insight into the long-term fate of NSC allocated to storage, and how new NSC (recent photosynthetic assimilates) mixes with old NSC (previously stored reserves) within the woody tissues of two temperate trees. In our previous analyses (Carbone *et al*., 2013; Richardson *et al*., 2013), in which we determined the mean age of NSC in a 2 cm stemwood core, we were unable to distinguish among candidate models for mixing of old and new reserves (Fig.1). Here, by extracting and aging NSC from individual growth rings in the stemwood, we have shown evidence for strong mixing of NSC across the youngest growth rings (where NSC concentrations are highest), but what we interpret to be much more limited ‘mixing in’ of younger NSC to older rings (where NSC concentrations are lower). Our results are consistent with the idea that while most of the NSC in trees is stored in a pool that is, well-mixed and turns over quickly, a substantial fraction of the reserves are much older and are not actively mixing with new assimilates.

Allocation and storage have long been recognized as an ‘Achilles’ Heel’ of simulation models (LeRoux & Lacointe, 2001), reflecting a general lack of understanding of these processes. Our results give support for a previously-hypothesized (Richardson *et al*., 2013) two-pool model representation of NSC reserves, with ‘fast’ and ‘slow’ cycling pools, which play functionally different roles in supporting growth and metabolism. Additionally, if the ‘fast’ and ‘slow’ cycling pools are physically distinct because they are stored in younger vs older rings, these results help to explain observations (Carbone *et al*., 2013) that NSC less than a year old is preferentially used for growth and metabolic demands, even when the mean age of NSC is much older. However, the slow pool is large enough that it cannot be ignored as a store of reserves. We conclude that it is overly simplistic to view (and for models to represent) NSC reserves as a single pool that is well mixed and turns over quickly.

## Supporting Information

**Table S1** Sugar and starch concentration data for white pine and red oak **Table S2** Radiocarbon data for white pine and red oak **Table S3** Tissue age, concentrations of extracted nonstructural carbon (NSC), and the radiocarbon (^14^C) age of extracted NSC, for roots and branches of a white pine and a red oak tree **Table S4** Woody biomass and nonstructural carbon (NSC) content in the stemwood of a white pine and red oak tree **Table S5** Woody biomass and nonstructural carbon (NSC) content in the root system of a white pine and red oak tree. **Table S6** Woody biomass and nonstructural carbon (NSC) content in the branches of a white pine and red oak tree **Methods S1** Uncertainty of radiocarbon measurements. **Methods S2** Allometric scaling from nonstructural carbon (NSC) concentrations to whole-tree budgets, and uncertainty characterization.
