# Supplementary material for: Distribution and mixing of old and new nonstructural carbon in two temperate trees
Source: New Phytol. 2015 Jan 5;206(2):590–7. doi: 10.1111/nph.13273 (PMC4405048; doi:10.1111/nph.13273)
Supplement: Supplementary file 1 — Table S1 Sugar and starch concentration data for white pine and red oak Table S2 Radiocarbon data for white pine and red oak Table S3 Tissue age, concentrations of extracted nonstructural carbon (NSC), and the radiocarbon (14C) age of extracted NSC, for roots and branches of a white pine and a red oak tree Table S4 Woody biomass and nonstructural carbon (NSC) content in the stemwood of a white pine and red oak tree Table S5 Woody biomass and nonstructural carbon (NSC) content in the root system of a white pine and red oak tree. Table S6 Woody biomass and nonstructural carbon (NSC) content in the branches of a white pine and red oak tree Methods S1 Uncertainty of radiocarbon measurements. Methods S2 Allometric scaling from nonstructural carbon (NSC) concentrations to whole-tree budgets, and uncertainty characterization. [file nph0206-0590-sd1.pdf]

New Phytologist **Supporting Information**

Distribution and mixing of old and new nonstructural carbon in two temperate trees

Authors: Andrew D. Richardson, Mariah S. Carbone, Brett A. Huggett, Morgan E. Furze, Claudia I. Czimczik, Jennifer C. Walker, Xiaomei Xu, Paul G. Schaberg and Paula Murakami

Article acceptance date: 09 December 2014

The following Supporting Information is available for this article:

Table S1 Sugar and starch concentration data for white pine and red oak

Table S2 Radiocarbon data for white pine and red oak

Table S3 Tissue age, concentrations of extracted nonstructural carbon (NSC), and the 14C age of extracted NSC, for roots and branches of a white pine and a red oak tree

Table S4 Woody biomass and nonstructural carbon (NSC) content in the stemwood of a white pine and red oak tree

Table S5 Woody biomass and nonstructural carbon (NSC) content in the root system of a white pine and red oak tree

Table S6 Woody biomass and nonstructural carbon (NSC) content in the branches of a white pine and red oak tree.

Methods S1 Uncertainty of radiocarbon measurements.

Methods S2 Allometric scaling from nonstructural carbon (NSC) concentrations to whole-tree budgets, and uncertainty characterization.

Table S1 Sugar and starch concentration data for (A) white pine and (B) red oak

(A) White pine	Sugars (mg g^{-1})	Starch (mg g^{-1})
Stemwood, 1 m height, Phloem	188.1 ± 24.1	26.5 ± 1.4
Stemwood, 1 m height, CY ring	33.5 ± 2.7	10.8 ± 1.4
Stemwood, 1 m height, 1 yr ring	13.3 ± 1.0	7.3 ± 0.7
Stemwood, 1 m height, 2 yr ring	10.4 ± 1.9	6.9 ± 2.1
Stemwood, 1 m height, 3 yr ring	10.5 ± 1.9	6.2 ± 1.1
Stemwood, 1 m height, 4 yr ring	6.0 ± 0.5	6.5 ± 1.9
Stemwood, 1 m height, 5 yr ring	5.6 ± 0.4	6.5 ± 1.0
Stemwood, 1 m height, 6 yr ring	7.8 ± 0.5	7.9 ± 2.5
Stemwood, 1 m height, 7 yr ring	7.8 ± 2.2	7.3 ± 0.9
Stemwood, 1 m height, 8 yr ring	2.1 ± 0.6	7.4 ± 0.8
Stemwood, 1 m height, 9 yr ring	13.1 ± 0.8	7.1 ± 0.2
Stemwood, 1 m height, 10 yr ring	6.3 ± 5.6	7.2 ± 0.4
Stemwood, 1 m height, 11 yr ring	5.2 ± 0.9	8.3 ± 1.5
Stemwood, 1 m height, 12 yr ring	9.3 ± 3.5	3.7 ± 0.6
Stemwood, 1 m height, 13 yr ring	12.5 ± 2.1	4.7 ± 2.3
Stemwood, 1 m height, 14 yr ring	10.4 ± 6.8	7.4 ± 0.8
Stemwood, 1 m height, 15 yr ring	10.0 ± 0.0	7.2 ± 1.5
Stemwood, 1 m height, 16 yr ring	8.4 ± 1.4	4.1 ± 0.7
Stemwood, 1 m height, 17 yr ring	4.7 ± 3.8	5.7 ± 2.3
Stemwood, 1 m height, 18 yr ring	4.4 ± 4.9	5.7 ± 1.0
Stemwood, 1 m height, 19–22 yr rings	4.9 ± 1.4	3.8 ± 1.2
Stemwood, 6 m height, CY ring	21.3 ± 2.5	6.6 ± 1.2
Stemwood, 6 m height, 1 yr ring	16.2 ± 3.0	4.7 ± 1.2
Stemwood, 6 m height, 2 yr ring	5.2 ± 2.1	6.5 ± 0.9
Stemwood, 6 m height, 3 yr ring	4.9 ± 0.7	6.5 ± 0.8
Stemwood, 6 m height, 4 yr ring	10.4 ± 0.0	8.3 ± 0.4
Stemwood, 6 m height, 5 yr ring	7.6 ± 2.9	4.6 ± 0.6
Stemwood, 6 m height, 6 yr ring	15.0 ± 0.4	4.4 ± 1.9
Stemwood, 6 m height, 7 yr ring	9.3 ± 3.6	4.0 ± 0.6
Stemwood, 6 m height, 8 yr ring	14.7 ± 1.1	6.5 ± 0.9
Stemwood, 6 m height, 9–14 yr rings	6.1 ± 0.4	4.8 ± 0.5
Stemwood, 10 m height, CY ring	15.7 ± 1.5	4.6 ± 1.2
Stemwood, 10 m height, 1 yr ring	21.6 ± 0.8	5.4 ± 0.1
Stemwood, 10 m height, 2 yr ring	20.2 ± 4.2	7.8 ± 1.2
Stemwood, 6 m height, 3–6 yr rings	10.9 ± 0.4	7.4 ± 2.0
Large coarse root, D1	4.3 ± 1.3	12.4 ± 0.9
Large coarse root, D2	5.1 ± 0.3	12.3 ± 0.7
Large coarse root, D3	7.8 ± 0.1	9.6 ± 4.0
Medium coarse roots	30.9 ± 0.6	21.7 ± 2.7
Fine roots	65.8 ± 3.4	69.0 ± 1.8
Current year branch wood	52.8 ± 3.0	7.6 ± 0.9
Multi-year branch wood	20.3 ± 4.2	9.7 ± 2.1

(B) Red oak	Sugars (mg g^{-1})	Starch (mg g^{-1})
Stemwood, 1 m height, Phloem	88.4 ± 1.6	8.7 ± 0.4
Stemwood, 1 m height, CY ring	38.9 ± 0.4	40.1 ± 4.4
Stemwood, 1 m height, 1 yr ring	40.1 ± 0.8	32.0 ± 2.7
Stemwood, 1 m height, 2 yr ring	48.1 ± 5.2	21.1 ± 1.7
Stemwood, 1 m height, 3 yr ring	44.8 ± 1.0	12.7 ± 1.6
Stemwood, 1 m height, 4 yr ring	39.9 ± 1.5	7.9 ± 1.5
Stemwood, 1 m height, 5 yr ring	37.0 ± 1.3	6.9 ± 1.6
Stemwood, 1 m height, 6 yr ring	38.2 ± 0.5	5.3 ± 1.0
Stemwood, 1 m height, 7 yr ring	33.7 ± 1.0	2.8 ± 0.5
Stemwood, 1 m height, 8 yr ring	10.4 ± 0.4	1.8 ± 0.8
Stemwood, 1 m height, 9 yr ring	4.9 ± 0.7	1.8 ± 1.1
Stemwood, 1 m height, 10 yr ring	5.4 ± 2.5	2.7 ± 0.9
Stemwood, 1 m height, 11 yr ring	10.9 ± 0.8	3.0 ± 1.1
Stemwood, 1 m height, 12 yr ring	8.7 ± 1.4	2.4 ± 1.5
Stemwood, 1 m height, 13 yr ring	14.8 ± 5.0	4.9 ± 1.7
Stemwood, 1 m height, 14 yr ring	7.9 ± 1.0	2.9 ± 0.6
Stemwood, 1 m height, 15 yr ring	9.2 ± 3.0	5.6 ± 0.8
Stemwood, 1 m height, 16 yr ring	10.2 ± 0.3	4.7 ± 0.5
Stemwood, 1 m height, 17 yr ring	6.2 ± 1.4	4.2 ± 1.3
Stemwood, 1 m height, 18 yr ring	16.0 ± 0.8	3.6 ± 0.4
Stemwood, 1 m height, 19 yr ring	11.7 ± 2.9	2.0 ± 0.7
Stemwood, 1 m height, 20 yr ring	6.6 ± 0.9	7.5 ± 2.1
Stemwood, 1 m height, 21–27 yr rings	5.2 ± 2.1	4.1 ± 0.5
Stemwood, 8 m height, CY and 1 yr rings	48.0 ± 3.9	46.8 ± 1.8
Stemwood, 8 m height, 2–3 yr rings	36.5 ± 0.6	22.4 ± 1.2
Stemwood, 8 m height, 4–5 yr rings	31.5 ± 5.5	12.2 ± 2.7
Stemwood, 8 m height, 6–-7 yr rings	17.4 ± 1.4	4.6 ± 1.4
Stemwood, 8 m height, 8–9 yr rings	8.1 ± 1.2	5.5 ± 1.1
Stemwood, 8 m height, 10–17 yr rings	7.1 ± 1.1	3.8 ± 1.2
Stemwood, 15 m height, CY and 1 yr rings	48.1 ± 1.0	48.0 ± 2.1
Stemwood, 15 m height, 2–3 yr rings	38.0 ± 0.6	29.1 ± 2.9
Stemwood, 15 m height, 4–5 yr rings	40.6 ± 1.4	13.0 ± 1.6
Stemwood, 15 m height, 6–10 yr rings	11.4 ± 2.9	3.1 ± 0.4
Large coarse root, D1	45.6 ± 0.7	149.8 ± 9.1
Large coarse root, D2	67.2 ± 3.8	154.1 ± 2.8
Large coarse root, D3	67.8 ± 2.9	117.1 ± 4.8
Medium coarse roots	47.1 ± 2.6	158.6 ± 6.6
Fine roots	45.6 ± 1.8	47.5 ± 1.8
Current year branch wood	78.7 ± 4.5	42.1 ± 3.3
Multi-year branch wood	59.8 ± 4.3	29.9 ± 2.3

Note: Values are mean ± 1 SD, where both are calculated across the three separate wells run on the plate reader for each extraction.

Table S2 Radiocarbon data for (A) white pine and (B) red oak

(A) White Pine		$\Delta^{14}C$ (‰)	Fraction Modern	^{14}C Age (yr)	Range (yr)
Sugars	Stemwood, 1 m height, CY ring	28.2 ± 2.8	1.0361 ± 0.0028	0.0	0.0 – 0.1
	Stemwood, 1 m height, 2 yr ring	33.3 ± 2.8	1.0412 ± 0.0028	0.6	0.0 – 1.3
	Stemwood, 1 m height, 4 yr ring	34.3 ± 2.7	1.0422 ± 0.0027	0.8	0.2 – 1.5
	Stemwood, 1 m height, 6 yr ring	39.9 ± 2.7	1.0479 ± 0.0027	2.3	1.5 – 3.2
	Stemwood, 1 m height, 8 yr ring	47.3 ± 2.7	1.0553 ± 0.0027	4.4	3.7 – 5.0
	Stemwood, 1 m height, 10 yr ring	54.6 ± 2.8	1.0627 ± 0.0028	6.0	5.4 – 7.2
	Stemwood, 1 m height, 14 yr ring	64.2 ± 2.9	1.0724 ± 0.0029	8.6	8.1 – 9.1
	Stemwood, 1 m height, 18 yr ring	78.9 ± 2.7	1.0873 ± 0.0027	11.0	10.6 – 11.4
	Large coarse root, D1	33.4 ± 2.7	1.0413 ± 0.0027	0.6	0.0 – 1.2
	Large coarse root, D2	38.6 ± 2.7	1.0466 ± 0.0027	1.9	1.2 – 2.8
	Large coarse root, D3	45.9 ± 2.8	1.0539 ± 0.0028	4.1	3.3 – 4.7
	Medium coarse roots	45.1 ± 2.8	1.0531 ± 0.0028	3.9	3.1 – 4.6
	Fine roots	41.6 ± 2.7	1.0496 ± 0.0027	2.9	2.0 – 3.7
	Current year branch wood	28.2 ± 2.8	1.0360 ± 0.0028	0.0	0.0 – 0.0
Starch	Stemwood, 1 m height, 2 yr ring	34.9 ± 2.9	1.0430 ± 0.0029	1.0	0.4 – 1.8
	Stemwood, 1 m height, 4 yr ring*	35.7 ± 2.8	1.0436 ± 0.0028	1.1	0.5 – 1.9
	Stemwood, 1 m height, 4 yr ring*	33.3 ± 2.7	1.0413 ± 0.0027	0.6	0.0 – 1.2
	Stemwood, 1 m height, 4 yr ring*	46.6 ± 2.7	1.0548 ± 0.0027	4.3	3.6 – 4.9
	Stemwood, 1 m height, 6 yr ring*	28.7 ± 1.7	1.0365 ± 0.0017	0.0	0.0 – 0.0
	Stemwood, 1 m height, 6 yr ring*	39.9 ± 2.7	1.0480 ± 0.0027	2.4	1.6 – 3.3
	Stemwood, 1 m height, 6 yr ring*	50.8 ± 3.0	1.0590 ± 0.0030	5.2	4.5 – 5.8
	Stemwood, 1 m height, 8 yr ring*	49.2 ± 2.7	1.0572 ± 0.0027	4.8	4.2 – 5.4
	Stemwood, 1 m height, 8 yr ring*	42.9 ± 2.0	1.0509 ± 0.0020	3.3	2.7 – 3.9
	Stemwood, 1 m height, 10 yr ring*	62.0 ± 2.8	1.0703 ± 0.0028	8.2	7.6 – 8.7
	Stemwood, 1 m height, 10 yr ring*	46.8 ± 2.8	1.0549 ± 0.0028	4.3	3.6 – 4.9
	Stemwood, 1 m height, 14 yr ring*	66.4 ± 1.7	1.0746 ± 0.0017	8.9	8.6 – 9.2
	Stemwood, 1 m height, 14 yr ring*	70.2 ± 1.8	1.0784 ± 0.0018	9.6	9.3 – 9.9
	Stemwood, 1 m height, 14 yr ring*	82.3 ± 2.8	1.0907 ± 0.0028	11.5	11.1 – 11.9
	Stemwood, 1 m height, 18 yr ring	95.5 ± 2.1	1.1039 ± 0.0021	13.8	13.4 – 14.5
	Large coarse root, D1*	31.9 ± 2.7	1.0398 ± 0.0027	0.3	0.0 – 0.9
	Large coarse root, D1*	36.9 ± 2.9	1.0449 ± 0.0029	1.5	0.8 – 2.3
	Large coarse root, D1*	35.4 ± 2.7	1.0435 ± 0.0027	1.1	0.5 – 1.8
	Large coarse root, D1*	34.9 ± 2.7	1.0430 ± 0.0027	1.0	0.4 – 1.7
	Large coarse root, D2*	52.2 ± 2.7	1.0604 ± 0.0027	5.5	4.9 – 6.2
	Large coarse root, D2*	50.7 ± 2.7	1.0589 ± 0.0027	5.2	4.6 – 5.8
	Large coarse root, D3*	58.0 ± 1.7	1.0661 ± 0.0017	7.4	7.0 – 7.8
	Large coarse root, D3*	63.0 ± 1.7	1.0711 ± 0.0017	8.4	8.1 – 8.7
	Medium coarse roots*	34.3 ± 2.7	1.0423 ± 0.0027	0.8	0.2 – 1.5
	Medium coarse roots*	37.7 ± 2.7	1.0457 ± 0.0027	1.7	1.0 – 2.5
Cellulose	Large coarse root, D1	39.7 ± 1.7	1.0476 ± 0.0017	2.2	1.7 – 2.7
	Large coarse root, D2	63.5 ± 1.9	1.0717 ± 0.0019	8.5	8.2 – 8.8
	Large coarse root, D3	96.1 ± 2.1	1.1045 ± 0.0021	13.9	13.5 – 14.7
	Medium coarse roots	48.0 ± 1.8	1.0560 ± 0.0018	4.6	4.2 – 5.0
	Fine roots	46.5 ± 1.7	1.0545 ± 0.0017	4.2	3.8 – 4.6

(B) Red oak		$\Delta^{14}C$ (‰)	Fraction Modern	^{14}C Age (yr)	Range (yr)
Sugars	Stemwood, 1 m height, CY ring	32.3 ± 2.7	1.0402 ± 0.0027	0.4	0.0 – 1.0
	Stemwood, 1 m height, 2 yr ring	32.1 ± 2.7	1.0400 ± 0.0027	0.3	0.0 – 0.9
	Stemwood, 1 m height, 4 yr ring	39.3 ± 2.9	1.0473 ± 0.0029	2.1	1.3 – 3.0
	Stemwood, 1 m height, 6 yr ring	46.9 ± 2.7	1.0549 ± 0.0027	4.3	3.6 – 4.9
	Stemwood, 1 m height, 8 yr ring	50.9 ± 2.8	1.0590 ± 0.0028	5.2	4.6 – 5.8
	Stemwood, 1 m height, 12 yr ring	71.4 ± 2.8	1.0796 ± 0.0028	9.8	9.3 – 10.3
	Stemwood, 1 m height, 16 yr ring	93.6 ± 2.9	1.1020 ± 0.0029	13.5	13.0 – 14.0
	Stemwood, 1 m height, 20 yr ring	115.4 ± 2.9	1.1240 ± 0.0029	17.8	17.3 – 18.3
	Large coarse root, D1	41.4 ± 2.8	1.0493 ± 0.0028	2.8	1.9 – 3.7
	Large coarse root, D2	61.8 ± 2.8	1.0700 ± 0.0028	8.2	7.6 – 8.7
	Large coarse root, D3	74.8 ± 2.8	1.0830 ± 0.0028	10.3	9.8 – 10.8
	Medium coarse roots	46.9 ± 2.8	1.0549 ± 0.0028	4.3	3.6 – 4.9
	Fine roots	59.1 ± 2.9	1.0672 ± 0.0029	7.6	6.9 – 8.2
	Current year branch wood	33.3 ± 2.8	1.0413 ± 0.0028	0.6	0.0 – 1.3
Starch	Stemwood, 1 m height, 6 yr ring*	34.0 ± 2.7	1.0419 ± 0.0027	0.7	0.1 – 1.4
	Stemwood, 1 m height, 6 yr ring*	44.5 ± 2.9	1.0527 ± 0.0029	3.8	2.9 – 4.5
	Stemwood, 1 m height, 8 yr ring*	35.0 ± 2.7	1.0429 ± 0.0027	0.9	0.3 – 1.6
	Stemwood, 1 m height, 8 y ring*	45.8 ± 2.9	1.0539 ± 0.0029	4.1	3.3 – 4.8
	Stemwood, 1 m height, 8 yr ring*	59.3 ± 3.1	1.0676 ± 0.0031	7.7	7.0 – 8.3
	Stemwood, 1 m height, 12 yr ring*	59.0 ± 2.8	1.0671 ± 0.0028	7.6	6.9 – 8.2
	Stemwood, 1 m height, 12 yr ring*	59.2 ± 1.6	1.0673 ± 0.0016	7.6	7.2 – 8.0
	Stemwood, 1 m height, 16 yr ring*	77.4 ± 3.2	1.0856 ± 0.0032	10.8	10.3 – 11.3
	Stemwood, 1 m height, 16 yr ring*	82.9 ± 1.8	1.0912 ± 0.0018	11.6	11.3 – 11.9
	Stemwood, 1 m height, 20 yr ring*	113.3 ± 3.1	1.1218 ± 0.0031	17.4	16.9 – 18.0
	Stemwood, 1 m height, 20 yr ring*	112.7 ± 2.1	1.1212 ± 0.0021	17.3	17.0 – 17.7
	Stemwood, 1 m height, 20 yr ring*	112.6 ± 1.8	1.1212 ± 0.0018	17.3	17.0 – 17.6
	Large coarse root, D2	52.2 ± 2.7	1.0602 ± 0.0027	5.5	4.9 – 6.1
	Large coarse root, D3*	62.9 ± 2.8	1.0710 ± 0.0028	8.3	7.8 – 8.8
	Large coarse root, D3*	83.2 ± 3.1	1.0916 ± 0.0031	11.7	11.3 – 12.2
	Medium coarse roots	29.9 ± 2.7	1.0378 ± 0.0027	0.0	0.0 – 0.4
	Fine roots	41.3 ± 2.9	1.0493 ± 0.0029	2.8	1.9 – 3.7
Cellulose	Large coarse root, D1	42.8 ± 1.8	1.0508 ± 0.0018	3.2	2.6 – 3.7
	Large coarse root, D2	96.2 ± 1.8	1.1046 ± 0.0018	14.0	13.7 – 14.7
	Large coarse root, D3	111.6 ± 2.5	1.1201 ± 0.0025	17.1	16.8 – 17.5
	Medium coarse roots	65.5 ± 1.8	1.0737 ± 0.0018	8.8	8.5 – 9.1
	Fine roots	71.4 ± 1.8	1.0796 ± 0.0018	9.8	9.5 – 10.1

Note: $\Delta^{14}C$ was measured at the W.M. Keck Carbon Cycle Accelerator Mass Spectrometry facility at UC Irvine. For convenience, we also report the corresponding value in Fraction Modern notation. Reported uncertainties are ± 1 SD of the analytical uncertainty as described in Methods S1. ^{14}C data were converted to age estimates using the bomb spike method as described in Methods. The reported age range is based on the analytical uncertainty. Asterisks denote samples for which multiple extractions were run independently.

Table S3 Tissue age, concentrations of extracted nonstructural carbon (NSC), and the 14C age of extracted NSC, for roots and branches of a white pine and a red oak tree

		Tissue	NSC Concentration (mg g^{-1})		NSC Age (years)	
**Species**	**Tissue type**	**Age (years)**	**Sugars**	**Starch**	**Sugars**	**Starch**
**White pine**	Large coarse root — D1	2.2 ± 0.5	4	12	0.6 ± 0.6	1.0 ± 0.8
	Large coarse root — D2	8.5 ± 0.3	5	12	1.9 ± 0.9	5.4 ± 0.7
	Large coarse root — D3	13.9 ± 0.8	8	10	4.1 ± 0.6	7.9 ± 0.4
	Medium coarse root	4.6 ± 0.4	31	22	3.9 ± 0.8	1.3 ± 0.8
	Fine roots	4.2 ± 0.4	66	69	2.9 ± 0.9	
	Curr.-year branches	0	53	8	0.0 ± 0.0	
	Multi-year branches	1+	20	10		
**Red oak**	Large coarse root — D1	3.2 ± 0.5	46	150	2.8 ± 0.9	
	Large coarse root — D2	14.0 ± 0.7	67	154	8.2 ± 0.5	5.5 ± 0.6
	Large coarse root — D3	17.1± 0.4	68	117	10.3 ± 0.5	10.0 ± 0.5
	Medium coarse root	8.8 ± 0.3	47	159	4.3 ± 0.7	0.0 ± 0.4
	Fine roots	9.8 ± 0.3	46	47	7.6 ± 0.7	2.8 ± 0.9
	Curr.-year branches	0	79	42	0.6 ± 0.6	
	Multi-year branches	1+	60	30		

Note: For all roots, tissue age was determined by ^{14}C analysis of extracted cellulose. For large coarse roots, D1–D3 denote root wood tissues of varying depth, from the youngest (most recently formed, or outermost = D1) to the oldest (D3) wood. Uncertainties represent 1 SD of the analytical uncertainty of the AMS measurements (see Methods S1).

Table S4 Woody biomass and nonstructural carbon (NSC) content in the stemwood of a white pine (top) and red oak (bottom) tree

				Aboveground Biomass			NSC in Ring Year		Ring Year as % of Total	
Species	Ring year	Ring width (mm)	Cumulative f (mm)	Cumulative (kg)	Ring Year (kg)	Ring as % of Total	Sugars (g)	Starch (g)	Sugars	Starch
**White pine**	1990	2.2	4	0	0.0	0	0	0	0	0
	1991	1.2	7	0	0.0	0	0	0	0	0
	1992	1.7	10	0	0.0	0	0	0	0	0
	1993	3.0	16	0	0.1	0	1	0	0	0
	1994	3.8	24	0	0.3	0	1	1	0	0
	1995	3.7	31	1	0.4	1	2	2	0	1
	1996	5.0	41	2	0.8	1	6	3	1	1
	1997	4.5	50	3	1.0	2	10	7	1	2
	1998	4.7	60	4	1.3	2	13	10	2	2
	1999	3.7	67	5	1.3	2	16	6	2	1
	2000	5.8	79	7	2.4	4	22	9	3	2
	2001	4.8	88	10	2.4	4	13	20	2	5
HW begins →	2002	4.5	97	13	2.7	4	17	19	3	4
	2003	4.8	107	16	3.2	5	42	23	7	5
	2004	4.5	116	19	3.4	6	7	26	1	6
	2005	4.9	126	23	4.2	7	33	31	5	7
	2006	4.2	134	27	4.0	7	31	31	5	7
	2007	4.0	142	32	4.2	7	23	27	4	6
	2008	4.9	152	37	5.5	9	33	36	5	8
	2009	4.6	161	43	5.8	10	61	36	9	8
	2010	5.1	171	50	6.9	11	71	47	11	11
	2011	3.8	179	55	5.5	9	74	40	11	9
	2012	3.2	185	60	5.0	8	167	54	26	13
	**Total**	92.6			60	100	644	428	100	100

				Aboveground Biomass			NSC in Ring Year		Ring Year as % of Total	
Species	Ring year	Ring width (mm)	Cumulative f (mm)	Cumulative (kg)	Ring Year (kg)	Ring as % of Total	Sugars (g)	Starch (g)	Sugars	Starch
**Red oak**	1983	1.5	3	0	0.0	0	0	0	0	0
	1984	0.5	4	0	0.0	0	0	0	0	0
	1985	1.3	7	0	0.0	0	0	0	0	0
	1986	1.6	10	0	0.0	0	0	0	0	0
	1987	0.7	11	0	0.0	0	0	0	0	0
	1988	0.8	13	0	0.0	0	0	0	0	0
	1989	1.3	16	0	0.1	0	0	0	0	0
	1990	2.3	20	0	0.2	0	1	1	0	0
	1991	1.7	24	1	0.2	0	1	1	0	0
	1992	3.2	30	1	0.4	1	3	3	0	0
	1993	3.3	36	2	0.6	1	7	1	0	0
	1994	2.8	42	2	0.7	1	11	3	1	0
	1995	2.9	48	3	0.9	1	5	4	0	0
	1996	3.8	55	5	1.4	2	14	7	1	1
	1997	3.0	61	6	1.3	2	12	7	1	1
	1998	4.0	69	8	2.1	3	16	6	1	1
	1999	2.8	75	10	1.6	2	24	8	1	1
	2000	3.6	82	12	2.4	3	21	6	1	1
	2001	2.7	87	14	2.0	3	22	6	1	1
	2002	2.9	93	16	2.3	3	13	6	1	1
	2003	3.5	100	20	3.2	4	16	6	1	1
HW begins →	2004	4.1	108	24	4.1	6	43	7	2	1
	2005	3.6	116	28	4.1	6	137	11	6	1
	2006	3.1	122	32	3.8	5	145	20	7	2
	2007	3.8	130	36	4.9	7	183	34	9	3
	2008	3.7	137	42	5.3	8	213	42	10	4
	2009	4.7	147	49	7.4	10	332	94	16	9
	2010	2.7	152	54	4.6	6	220	97	10	10
	2011	4.1	160	61	7.3	10	293	234	14	23
	2012	5.0	170	71	9.8	14	380	392	18	39
	**Total**	85.1			71	100	2115	997	100	100

Note: See Methods S2 for details of allometric calculations. Biomass is reported in terms of oven-dry wood. NSC is reported in g of sugars and starch. 'HW begins' denotes the ring in which dark-colored heartwood is first visible.

Table S5 Woody biomass and nonstructural carbon (NSC) content in the root system of a white pine (top) and red oak (bottom) tree

**Species**					**NSC in biomass (g)**	
**White pine**			**Fraction of coarse root biomass**	**Biomass (kg)**	**Sugars**	**Starch**
**Coarse roots**	Scenario 1	Large roots	90%	18.9	107	202
		Intermediate roots	10%	2.1	65	46
		Total		21.0	172	248
	Scenario 2	Large roots	75%	15.8	89	168
		Intermediate roots	25%	5.3	163	116
		Total		21.0	252	284
	Scenario 2	Large roots	50%	10.5	60	112
		Intermediate roots	50%	10.5	326	231
		Total		21.0	385	343
**Fine roots**	Scenario 1	Low fine roots	5%	1.1	69	72
	Scenario 2	Middle fine roots	15%	3.2	208	217
	Scenario 3	High fine roots	30%	6.3	416	435
**Total roots**	Scenario 2	Large/Intermediate/Fine	75%/25%/15%	24.2	460	501

**Red oak**					**NSC in biomass (g)**	
			**Fraction of coarse root biomass**	**Biomass (kg)**	**Sugars**	**Starch**
**Coarse roots**	Scenario 1	Large roots	90%	23.1	1392	3237
		Intermediate roots	10%	2.6	120	407
		Total		25.6	1512	3644
	Scenario 2	Large roots	75%	19.2	1160	2697
		Intermediate roots	25%	6.4	301	1019
		Total		25.6	1461	3716
	Scenario 2	Large roots	50%	12.8	773	1798
		Intermediate roots	50%	12.8	602	2037
		Total		25.6	1375	3836
**Fine roots**	Scenario 1	Low fine roots	5%	1.3	59	60
	Scenario 2	Middle fine roots	15%	3.8	177	181
	Scenario 3	High fine roots	30%	7.7	354	361
**Total roots**	Scenario 2	Large/Intermediate/Fine	75%/25%/15%	29.5	1638	3897

Note: Biomass is reported in terms of oven-dry wood. NSC is reported in g of sugars and starch. For both large and intermediate coarse roots, and fine roots, Scenarios 1–3 denote a range of plausible assumptions about the relative distribution of different sized roots, and are intended to bracket the likely uncertainty (see Methods S2).

Table S6 Woody biomass and nonstructural carbon (NSC) content in the branches of a white pine (top) and red oak (bottom) tree

Species			
**White pine**		**Branch wood biomass (kg)**	
	Current year	1.6	
	Previous years	17.2	
	Total	18.8	
		**NSC in branch wood (g)**	
		**Sugars**	**Starch**
	Current year	82	12
	Previous years	344	172
	Total	427	185
**Red oak**		**Branch wood biomass (kg)**	
	Current year	6.0	
	Previous years	37.7	
	Total	43.8	
		**NSC in branch wood (g)**	
		**Sugars**	**Starch**
	Current year	320	48
	Previous years	755	377
	Total	1075	426

Note: Biomass is reported in terms of oven-dry wood. NSC is reported in g of sugars and starch.

Methods S1 Uncertainty of radiocarbon measurements.

We assessed the uncertainty in the ^{14}C measurements in several ways. First, there is an analytical uncertainty (1 SD) determined for each sample run on the AMS. This is derived based on counting statistics and the analytical reproducibility of the primary standards (Oxalic acid standard OXI) run with each wheel (Stuiver *et al*., 1977). For the samples analyzed here, the average uncertainty (mean ±1 SD) in measured $\Delta^{14}C$ is 2.5 ±0.5‰ (n = 82). Over the last 5 y, the mean rate of decline in atmospheric ^{14}C has been 3.7‰ per year. Thus, the AMS uncertainty translates to an uncertainty of about 0.6 ±0.2 y in ^{14}C-based age estimates.

Second, secondary standards were run with each wheel. For our samples, we measured a mean (±1 SD) $\Delta^{14}C$ value of 489.5 ±3.6‰ (n = 4) for ANU sucrose (IAEA C-6), which corresponds to 1.5010 ±0.0037 in Fraction Modern notation, and which compares favorably to the published (Xu *et al*., 2010) long-term mean (±1 SE) value measured at UCI of 1.5016 ±0.0002 (approximately 490.0‰, when back-corrected to 2014 from Fraction Modern).

Third, to evaluate our extraction method, we compared the $\Delta^{14}C$ of sugars and starch extracted from rye and wheat standards with the $\Delta^{14}C$ of bulk tissue for these same standards. For both sugars ($\Delta\Delta^{14}C$ = 0.8 ± 3.7‰, n = 6) and starch ($\Delta\Delta^{14}C$ = –2.6 ± 2.4‰), the deviations are essentially within the AMS analytical uncertainty. We also did the same for sugars extracted from our annual plant samples ($\Delta\Delta^{14}C$ = 0.3 ± 2.2‰, n = 3), confirming the results for rye and wheat.

Finally, for some of our starch samples, we conducted multiple extractions and ran these separately on the AMS. Relative to the multi-sample mean for a given tissue, these had a standard deviation of ±5.8‰ (n = 37). Note that this value incorporates uncertainty due to natural variability among samples and also uncertainty associated with the extraction procedure, as well as the inherent AMS analytical uncertainty. As a conservative estimate of overall uncertainty, this is about equal to 1.5 times the annual decline in atmospheric ^{14}C in recent years.

Also, we remind the reader that the measured ^{14}C age is the *mean* age of C in that sample. A given sample with a radiocarbon age of *x* years could have all been fixed from the

atmosphere *x* years previously. Alternatively, the same radiocarbon age could be measured from a sample that is comprised partially of carbon with an age of *x* years, as well as some carbon which is (much) older, and some carbon which is (much) younger. We acknowledge that the method cannot distinguish between these scenarios.

References

Stuiver M, Polach. HA. **1977**. Reporting of ^{14}C data. *Radiocarbon* **19**: 355–363.

Xu X, Khosh M, Druffel-Rodriguez K, Trumbore S, Southon J. **2010**. Is the consensus value of ANU sucrose (IAEA C-6) too high? *Radiocarbon* **52**: 866–874.

Methods S2 Allometric scaling from nonstructural carbon (NSC) concentrations to whole-tree budgets, and uncertainty characterization.

Allometric Scaling

Following Jenkins *et al.* (2004), we calculated total above-ground biomass, B (kg), as a function of DBH (cm):

$$B = \exp(\beta_0 + \beta_1 \ln \text{DBH})$$

From their Table 1, we used generic pine species (*Pinus* spp.) parameters for the white pine (β_0 = –2.5356, β_1 = 2.4349), and generic hardwood species (designated "maple/oak/hickory/beech") parameters (β_0 = –2.4800, β_1 = 2.4835) for the red oak, to calculate B.

In Jenkins *et al.* (2004), B includes foliage, stemwood, bark, and branches. We calculated the fraction (f) of B corresponding to each of the first three components, using values of α_0 and α_1 from their Table 2, as:

$$f = \exp(\alpha_0 + \alpha_1 / \text{DBH})$$

For example, for the pine, we used α_0 = –0.3737 and α_1 = –1.8055 to determine the stemwood fraction. Given that the tree we sampled had DBH = 18.5 cm, we estimated the stemwood fraction to be 0.624 of B. By a similar calculation, we estimated the stemwood fraction of the oak (α_0 = –0.3065 and α_1 = –5.4240; DBH = 17.0 cm) to be 0.535.

We determined the branch fraction by difference, i.e.:

$$f_{\text{branch}} = 1 - f_{\text{foliage}} - f_{\text{stemwood}} - f_{\text{bark}}$$

This gave us values for f_{branch} of 0.19 for the pine and 0.33 for the oak. To partition branch biomass to current-year and multi-year growth, we assumed that the ratio of (current year branch wood)/(total branch wood) was the same as (current year stemwood)/(total stemwood). By this estimate, current year branch wood accounted for 8% of the total branch wood for the pine, and 14% of the total branch wood for the oak.

Jenkins *et al.* (2004) used a similar proportional scaling approach to estimate coarse root biomass as a function of B. For the pine, we calculated $f_{\text{coarse roots}}$ = 0.22, compared to 0.19 for

the oak. These estimates are consistent with a root-to-shoot ratio of 1:5, which is often typical for temperate forest trees.

Jenkins *et al.* (2004) did not report equations or coefficients for partitioning the coarse root biomass to large and intermediate diameter roots. We therefore made three calculations, intended to bracket a range of plausible situations: (1) assuming that large roots comprised 90% of the coarse root pool; (2) assuming that large roots comprised 75% of the coarse root pool; and (3) assuming that large and intermediate roots were each 50% of the coarse root pool.

Similarly, Jenkins *et al.* (2004) did not report equations or coefficients for estimating fine root biomass. Thus, we again made three calculations to bracket a range of plausible situations: (1) assuming that fine roots were an additional 5% of coarse root biomass; (2) assuming that fine roots were an additional 15% of coarse root biomass; and (2) assuming that fine roots were an additional 30% of coarse root biomass.

Uncertainty characterization

As acknowledged already, our analysis was based on allometric equations for generic pine species and generic hardwood species. We did this because Jenkins *et al.* (2004) did not consistently report species-specific regression coefficients by which to partition total tree biomass to above- and below-ground biomass components for the tree species we focused on here, in our geographic region.

That said, we used the data in Jenkins *et al.* (2004) to characterize the uncertainty in allometric scaling for the specific species of interest. Across six pine studies in Jenkins *et al.* (2004), the mean above-ground biomass of a 20 cm pine is estimated to be 91 ±28 kg (mean ±1 SD), compared with 116 kg based on the generic pine equation we used. Thus, we may be over-estimating biomass (and hence NSC pool sizes, which scale with biomass) by about 25%. In those studies that reported it, the branch fraction is 0.17 ±0.5, which is close to the value we used (0.19); the coarse root fraction is 0.22 (one study), identical to the value we used. Based on this analysis, we conclude that for pine the main source of uncertainty is total biomass, rather than partitioning to different biomass components.

For seven oak studies in Jenkins *et al.* (2004), the mean above-ground biomass of a 20 cm oak is estimated to be 217 ±41 kg (mean ±1 SD), compared with 196 kg based on the generic hardwood equation we used. Thus, we may be under-estimating tree biomass by about 5-10%. In those studies that reported it, the branch fraction is 0.19 ±0.10, which is 40% lower than the value we used (0.33). There are no published allometric equations for oak root biomass. Therefore, in contrast to pine, for oak the main source of uncertainty in this analysis is the partitioning to different biomass components.

Reference

Jenkins JC, Chojnacky DC, Heath LS, Birdsey RA. **2004**. *Comprehensive database of diameter-based biomass regressions for North American tree species*. USDA Forest Service, Northeastern Research Station, Newtown Square, PA, USA.
